# Does Prior Nasal Airway Surgery Impact Hypoglossal Nerve Stimulation for Obstructive Sleep Apnea?

**DOI:** 10.1002/oto2.70008

**Published:** 2024-12-16

**Authors:** Vaibhav H. Ramprasad, Anna Matzke, Lauren Makey, Eugene Chio, Armin Steffen, Joachim T. Maurer, Clemens Heiser, Ryan J. Soose

**Affiliations:** ^1^ Cleveland Clinic Head and Neck Institute Cleveland OH USA; ^2^ Inspire Medical Systems Inc. Minneapolis Minnesota USA; ^3^ The Ohio State University Columbus OH USA; ^4^ ENT Sleep Laboratory University of Luebeck Luebeck Germany; ^5^ Universitäts‐klinikum Mannheim, Universität Heidelberg Mannheim Germany; ^6^ Klinikum Rechts der Isar Technische Universität München Munich Germany; ^7^ Department of Otolaryngology University of Pittsburgh Medical Center Pittsburgh PA USA

**Keywords:** hypoglossal nerve stimulation, nasal surgery, obstructive sleep apnea

## Abstract

**Objective:**

Nasal surgery can improve patient‐reported obstructive sleep apnea (OSA) outcomes as well as adherence with medical device treatments. The aim of this study was to examine whether previous nasal surgery was associated with hypoglossal nerve stimulation (HNS) therapy outcomes.

**Study Design:**

Retrospective observational cohort study was performed utilizing the multicenter international HNS registry (ADHERE).

**Methods:**

Propensity score matching generated a cohort of HNS patients with prior nasal surgery (NS) and a comparable cohort without prior nasal surgery (WNS). Data included demographics and therapy outcome measures including apnea‐hypopnea index (AHI), Epworth Sleepiness Scale (ESS), therapy use, and responder rate. Student's *t*‐test was used to compare normally‐distributed numeric data, Fisher's exact test to compare categorical data, and 1‐sided *t*‐tests to determine noninferiority.

**Results:**

From the ADHERE dataset, 169 HNS patients were identified and matched from each cohort. AHI reduction was 21.01 ± 17.94 in the WNS cohort and 18.39 ± 16.4 in the NS cohort (*P* = .162). ESS reduction in the WNS cohort was 4.85 ± 4.98 and 4.48 ± 5.83 in the NS cohort (*P* = .528). Therapy use was similar, 5.67 ± 1.95 in WNS and 5.97 ± 2.06 in NS (*P* = .181). Responder rate was also similar in WNS (64.5%) and NS (62.1%) groups (*P* = .735).

**Conclusion:**

Prior nasal surgery was not a predictor of HNS therapy response or adherence. Future prospective studies of HNS candidates with nasal airway obstruction may better determine the role of adjunctive nasal surgery in this population.

Obstructive sleep apnea (OSA) is a breathing disorder related to the pathologic collapse of the upper airway resulting in apnea during sleep. It has traditionally been treated with Continuous Positive Airway Pressure (CPAP). More recently, hypoglossal nerve stimulation (HNS) has been utilized as an alternative therapy for OSA patients who are unable to adhere to CPAP. HNS therapy results in contraction of the genioglossus muscle which serves as a pharyngeal dilator by protruding the tongue.^1^ HNS therapy has been shown to enlarge the retroglossal and retropalatal airway, which is thought to be due to passive coupling with the palatoglossus muscle.^2^

Nasal obstruction is known to play a role in the pathophysiology of OSA through a variety of mechanisms. Change in airflow and resistance at the level of the nose can lead to negative intraluminal pressure downstream leading to collapse of the oropharynx exacerbating underlying OSA. Furthermore, nasal obstruction inherently leads to increased oral breathing which has been shown to have 2.5 times higher resistance resulting in narrowing of the pharyngeal lumen and posterior collapse of the base of tongue. Multiple mechanisms have been implicated in the role of the nasal airway in mediating nocturnal respiration.^3^, ^4^, ^5^, ^6^

The role of nasal airway obstruction on treatment‐related outcomes of OSA is an area of active study. In a large cohort study of OSA patients, 65% of treatment naïve OSA patients were identified to have nasal obstruction symptoms. Nocturnal nasal obstruction was associated with daytime sleepiness and reduced mental health.^7^ Nasal function has been inversely associated with CPAP compliance as nasal resistance has been shown to be significantly higher in patients who discontinue CPAP therapy after initiation.^8^ Nasal surgery for nasal obstruction has showed inconsistent results in changing the apnea‐hypopnea index (AHI).^9^, ^10^, ^11^, ^12^ Surgical therapy to lower nasal resistance, however, has been strongly correlated with improvement in patient‐reported outcome measures such as reduced snoring, improved Epworth sleepiness score (ESS), and improved patient‐reported sleep quality. Though polysomnography measures may not change, patients undergoing nasal surgery are more likely to show lower pressure requirements and improved CPAP adherence, with a significant proportion of patients converting from non‐adherent to CPAP to adherent to CPAP.^13^, ^14^, ^15^, ^16^ Similar associations between nasal obstruction and medical device outcomes have been shown in patients utilizing oral appliance therapy (OAT). OAT nonresponders had significantly higher nasal resistance at baseline compared to OAT responders.^17^

The role of prior pharyngeal and palate surgery has not been shown to consistently impact HNS outcomes. Multiple retrospective studies have compared outcomes between patients undergoing HNS with no prior pharyngeal surgery and HNS patients with prior pharyngeal surgery (primarily uvulopalatopharyngoplasty and related modifications). No difference has been demonstrated in both objective (AHI, responder rate) and subjective (ESS) measures.^18^, ^19^, ^20^

Despite extensive literature on the role of nasal airway surgery on OSA, its role in HNS has not been previously described. The aim of this study was to examine whether prior nasal surgery was associated with HNS treatment efficacy.

## Methods

### Participants

The ADHERE registry, a large multicenter prospective registry of patients undergoing HNS implantation, was utilized to generate cohorts of HNS patients with and without prior nasal surgery. The ADHERE registry collects data including demographics, polysomnographic and patient‐reported OSA outcomes, surgical complications, and therapy adherence. The registry includes adults with a history of moderate‐to‐severe OSA (AHI 15‐65 events/h) with inadequate CPAP adherence, and an absence of complete concentric retropalatal collapse on drug‐induced sleep endoscopy (DISE). Patients with prior nasal surgery (NS) and patients without prior nasal surgery (WNS) were matched for comparison based on demographic variables, baseline BMI, prior CPAP use or Oral Appliance Therapy (OAT), baseline AHI, and baseline ESS (Epworth Sleepiness Scale). Further data was collected on the type of nasal surgery that was reported by the NS group (eg, septoplasty, turbinate reduction, polyp removal). Data on therapy outcome measures including AHI (as assessed by post‐operative polysomnography or home sleep test), ESS, therapy use (h/day), and responder rate at final follow up was collected. Responder rate was defined as AHI <20 and ≥50% AHI reduction in postoperative period.

### IRB Review

The ADHERE registry was reviewed by each site's institutional review board or received ethics committee and received approval. All study participants provided written informed consent. The study was registered with http://www.clinicaltrials.gov, NCT02907398.

### Statistical Methods

This was a retrospective analysis of ADHERE registry data. A cohort of HNS patients with prior nasal surgery (NS) and a comparable cohort without prior nasal surgery (WNS) were generated using 1:1 propensity score matching using the nearest neighbor method. Patients were matched based on the following variables: prior oral appliance, prior CPAP use, Gender, Age, baseline BMI, baseline AHI, Baseline ESS. Only patients with a complete dataset were included in final analysis and all patients had ESS, AHI, and therapy use at final follow‐up. Unless otherwise specified, Student's *t*‐test for numeric variables and Fisher's exact tests for categorical variables were used to detect a difference in outcome measures between the NS and WNS on final follow up.

One‐sided *t*‐test at the 2.5% significance level with noninferiority margin of 7.5 events/h for AHI, 2 points for ESS and 0.5 h/night for therapy use were performed to determine noninferiority.

## Results

A total of 751 of 3327 (22.6%) patients in the ADHERE registry had undergone a nasal surgical procedure prior to HNS implantation. Further details regarding the type of procedure performed are described in Table 1.

**Table 1:** Nasal Procedure Type and Frequency

Type of surgery(s)	Frequency
Nasal procedure	751
Septoplasty	308
Turbinate surgery and Septoplasty	129
Other nasal	97
Turbinate surgery	56
Other nasal and septoplasty	50
Polyp removal	29
Other nasal and turbinate surgery and septoplasty	22
Other nasal and turbinate surgery	7
Polyp removal and septoplasty	6
Other nasal and polyp removal and septoplasty	3
Other nasal and polyp removal	2
Polyp removal and turbinate surgery	1
Polyp removal and turbinate surgery and septoplasty	1

Participant demographic and clinical variables for both the cohort with prior nasal surgery (NS) and without prior nasal surgery (WNS) are described in Table 2. Majority of patients in the WNS cohort (n = 2576) were male (70.6%) and Caucasian (92.9%). This was similar to the NS cohort (n = 751), which had majority male (76.7%) and Caucasian (92.8%) patients. In comparing patient demographics between the 2 cohorts, prior to matching, the NS cohort had a lower age (58.17 ± 11.45) than the WNS cohort (61.04 ± 12.68). The NS cohort also had a lower proportion of female patients (23.3%) compared to the WNS cohort (29.4%). The NS cohort (27%) also reported a higher incidence of preoperative oral appliance trial compared to the WNS cohort (20.3%).

**Table 2:** Demographic and Baseline Data for the Entire Cohort (n* =* 3327)

Variable	No nasal surgery	Nasal surgery	*P* value
N	2576 (77.4%)	751 (22.6%)	
Age	61.04 ± 12.68 (62) N = 2470	58.17 ± 11.45 (58) N = 715	<.001
AHI‐Baseline	34.92 ± 15.45 (32) N = 2488	35.04 ± 15.99 (32.4) N = 715	.865
BMI‐Baseline	29.28 ± 3.73 (29.37) N = 2447	29.2 ± 3.81 (29.35) N = 695	.618
ESS‐Baseline	11.09 ± 5.53 (11) N = 2198	11.31 ± 5.55 (12) N = 615	.389
Female	29.4% (755)	23.3% (174)	.001
White	92.9% (2392)	92.8% (697)	.936
Hispanic or Latino	2.1% (52)	3.4% (25)	.052
Prior oral app use	20.3% (524)	27% (203)	<.001
Prior CPAP use	96.2% (2479)	97.5% (732)	.114

Format for numeric variables: mean ± SD (median).

Patients from both the WNS and the NS groups were matched with 169 patients in each cohort. In the matched groups, patient demographic and baseline variables were similar with no statistically significant difference between the WNS and NS cohort (Table 3). The responder rate was similar between the WNS cohort (64.5%) and the NS cohort (62.1%). Both cohorts also revealed no difference in final posttreatment AHI (14.61 ± 14.25 in the WNS cohort and 15.8 ± 13.95 in the NS cohort) and post‐treatment ESS (7.61 ± 4.88 in the WNS cohort and 7.47 ± 4.76 in the NS cohort). Therapy use was also similar in both cohorts (5.67 ± 1.95 h/night in WNS cohort and 5.97 ± 2.06 h/night in the NS cohort) (Table 4).

**Table 3:** Demographics and Baseline Data for Matched Patients

Variable	No nasal surgery	Nasal surgery	*P* value
N	169 (50%)	169 (50%)	
Age	58.21 ± 10.71 (58)	57.57 ± 10.44 (58)	.579
AHI‐Baseline	35.62 ± 14.61 (32.7)	34.19 ± 14.1 (31)	.359
BMI‐Baseline	29.08 ± 3.5 (29.37)	29.33 ± 4.3 (29.29)	.555
ESS‐Baseline	12.46 ± 5.38 (13)	11.95 ± 5.36 (12)	.384
Female	15.4% (26)	17.8% (30)	.661
White	93.5% (158)	97.6% (165)	.110
Hispanic or Latino	2.4% (4)	0.6% (1)	.213
Prior oral app use	28.4% (48)	27.2% (46)	.903
Prior CPAP use	99.4% (168)	98.8% (167)	1.000

Format for numeric variables: mean ± SD (median).

**Table 4:** Outcomes Data for Matched Patients

Variable	No nasal surgery (n = 169)	Nasal surgery (n = 169)	*P* value
Final AHI^a^	14.61 ± 14.25 (12)	15.8 ± 13.95 (12)	.294
Decrease in AHI	21.01 ± 17.94 (19.4)	18.39 ± 16.4 (16.7)	.162
Final ESS^a^	7.61 ± 4.88 (7)	7.47 ± 4.76 (7)	.776
Decrease in ESS	4.85 ± 4.98 (4)	4.48 ± 5.83 (4)	.528
Therapy use	5.67 ± 1.95 (5.71)	5.97 ± 2.06 (6.14)	.181
Nonresponder	35.5% (60)	37.9% (64)	.735
Responder	64.5% (109)	62.1% (105)	.735

Format for numeric variables: mean ± SD (median).

^a^ *P*‐value derived from Wilcoxon rank‐sum test.

To further assess HNS outcomes between the NS cohort and WNS cohort, noninferiority analysis was conducted on change in AHI and ESS in postoperative setting. An accepted lower bound for change in AHI was set at −7.5 and for ESS was set at −2. The change in AHI and ESS and therapy use in NS patients was shown to be noninferior in postoperative setting compared to patients in the WNS cohort (5, 6, 7).

**Table 5:** Noninferiority Analysis for Change in AHI from Baseline to Final Follow‐Up

Group	Change in AHI (mean ± SD)	N	Mean difference	Lower bound of confidence interval	Accepted lower bound	*P* value
No nasal surgery	21.01 ± 17.94	169	−2.62	−6.3	−7.5	.0047341
Nasal surgery	18.39 ± 16.4					.0047341

**Table 6:** Noninferiority Analysis for Change in ESS from Baseline to Final Follow‐Up

Group	Change in ESS (mean ± SD)	N	Mean difference	Lower bound of confidence interval	Accepted lower bound	*P* value
No nasal surgery	4.85 ± 4.98	169	−0.37	−1.53	−2	.0030737
Nasal surgery	4.48 ± 5.83					.0030737

**Table 7:** Noninferiority Analysis for Therapy Use at Final Follow‐Up

Group	Therapy use in hours (mean ± SD)	N	Mean difference	Lower bound of confidence interval	Accepted lower bound	*P* value
No nasal surgery	5.67 ± 1.95	169	0.29	−0.14	−0.5	.0001644
Nasal surgery	5.97 ± 2.06					.0001644

## Discussion

Increased nasal resistance is a known pathophysiologic contributor to upper airway collapsibility during sleep through multiple mechanisms, including disruption of the nasal‐ventilatory reflex, unfavorable mouth opening and jaw position, and increased negative intraluminal pressure in the pharynx.^21^ Although surgical treatment of nasal airway obstruction in patients with OSA has not been shown to consistently improve AHI, nasal surgery has been shown to improve snoring, patient‐reported sleep quality, and daytime sleepiness.^11^, ^16^, ^22^, ^23^ Furthermore, nasal airway surgery has been shown to improve CPAP adherence and lower CPAP pressure requirements.^8^, ^10^, ^24^

The downstream upper airway effects of nasal airway obstruction have also been demonstrated on DISE.^25^ Bosco et al demonstrated change in upper airway collapsibility in patients undergoing DISE before and after nasal airway surgery. Prior to nasal surgery, 74% of patients demonstrated multilevel obstruction at the oropharynx and hypopharynx. After nasal surgery, only 50% showed multilevel collapse. The number of patients without collapse of the oropharynx increased. These results suggest that nasal airway surgery may modify the extent and pattern of pharyngeal airway collapse, specifically in the oropharynx, which can directly impact HNS candidacy and efficacy.

Pang et al, reviewed the role of nasal surgery in outcomes of multilevel upper airway surgery for OSA. Their study demonstrated greater reduction in AHI, higher percentage change in ESS, and increased success rate of surgery for those with nasal obstruction undergoing concomitant nasal surgery and multilevel pharyngeal surgery compared to those undergoing multilevel pharyngeal surgery alone (68.2% vs 55.0%). They concluded that patients with severe nasal obstruction could potentially have more severe upper airway obstruction with more negative pharyngeal pressure leading to worse airway collapse hence improved outcomes in patients with concomitant nasal surgery.^26^

HNS provides an alternative for the treatment of patients with OSA that are intolerant of CPAP. This is the largest study examining the association between prior nasal airway surgery and outcomes of HNS for OSA. The ADHERE registry incorporates the real‐life clinical experience (outside of a trial setting) of dozens of centers leading to a robust HNS cohort. In our study, prior nasal airway surgery was not associated with HNS effectiveness or therapy adherence. Furthermore, outcomes in patients with prior nasal airway surgery were noninferior to those patients without nasal surgery. This suggests that despite potential implications of nasal obstruction on upper airway collapsibility, patients with or without a history of prior nasal surgery can be counseled preoperatively with the same expectations with regard to HNS outcomes.

Although this study is the largest of its kind evaluating nasal airway surgery and HNS, it does have limitations which temper conclusions that can be drawn from this data. First, this study included retrospectively collected data and only patients that had nasal surgery prior to HNS were analyzed. With this analysis and methodology, we were unable to determine whether those patients with nasal surgery had persistent nasal obstruction postoperatively. Presumably, these patients had successful surgery with correction of nasal obstruction prior to HNS. However, since the status of the nasal airway prior to implantation was not documented, we cannot directly evaluate the effects of nasal obstruction on HNS. Additionally, our study was limited by the extent of data collected through the ADHERE registry. While the registry recorded type of nasal surgery, it did not record the extent or outcomes of the nasal surgery, nor did it record the current status of the patients’ nasal airway. Furthermore, we are unable to account for variation in clinical judgment and surgical technique of nasal airway surgery prior to HNS implantation in our cohort.

In summary, our study explored the effect of a documented history of prior nasal surgery on HNS outcomes. We demonstrated no difference in outcomes between patients with prior nasal surgery and those without prior nasal surgery. Patients with symptomatic nasal obstruction, however, particularly those struggling with incomplete hypoglossal HNS response may benefit from adjunctive nasal surgery and further prospective research is needed to evaluate the role of nasal airway obstruction and adjunctive nasal surgery in long‐term HNS outcomes.

## Author Contributions

**Vaibhav H. Ramprasad**, Made substantial contribution to design of work, acquisition, analysis and interpretation of data; drafted article and revised critically for intellectual content; approved manuscript for publication; **Anna Matzke**, Made substantial contribution to acquisition, analysis and interpretation of data; drafted article and revised critically for intellectual content; approved manuscript for publication; **Lauren Makey**, Made substantial contribution to acquisition, analysis and interpretation of data; drafted article and revised critically for intellectual content; approved manuscript for publication; **Eugene Chio**, Made substantial contribution to analysis and interpretation of data; critically revised article for intellectual content; approved manuscript for publication; **Armin Steffen**, Made substantial contribution to analysis and interpretation of data; critically revised article for intellectual content; approved manuscript for publication; **Joachim T. Maurer**, Made substantial contribution to analysis and interpretation of data; critically revised article for intellectual content; approved manuscript for publication; **Clemens Heiser**, Made substantial contribution to analysis and interpretation of data; critically revised article for intellectual content; approved manuscript for publication; **Ryan J. Soose**, Made substantial contribution to design of work, acquisition, analysis and interpretation of data; drafted article and revised critically for intellectual content; approved manuscript for publication.

## Disclosure

### Competing interests

Ramprasad: None. Matzke: Employed by Inspire Medical Systems. Makey: Employed by Inspire Medical Systems. Chio: Consulting with Inspire Medical. Steffen: Consulting with Inspire Medical and Zoll Respicardia. Maurer: Institutional research support from LivaNova Medical, MedEl, Inspire Medical; Consulting and proctoring with Nyxoah; invited lectures with Inspire Medical, Neuwirth Medical, XM‐Consulting. Heiser: Institutional research support from Inspire Medical and Nyxoah. Soose: Institutional research support from Inspire Medical; Consulting with Cryosa Inc, Inspire Medical, and XII Medical.

### Funding source

None.
